# Antifungal Activity of Chitosan Polymeric Nanoparticles and Correlation with Their pH Against *Mucor circinelloides* Causing Mucormycosis, Along with *Penicillium notatum* and Aspergillus Species

**DOI:** 10.1007/s00284-023-03555-y

**Published:** 2023-12-22

**Authors:** Fatma I. Abo El-Ela, Walid Hamdy Hassan, Alaa M. Amer, S. I. El-Dek

**Affiliations:** 1 Department of Pharmacology, Faculty of Veterinary Medicine, Beni-Suef University, Beni-Suef, 62511 Egypt; 2 Department of Microbiology, Mycology and Immunology, Faculty of Veterinary Medicine, Beni-Suef University, Beni-Suef, 62511 Egypt; 3 Materials Science and Nanotechnology Department, Faculty of Postgraduate Studies for Advanced Sciences, Beni-Suef University, Beni-Suef, 62511 Egypt

## Abstract

Mucormycosis is uncommon, yet it is more prevalent among individuals with underlying health conditions and those who are immunocompromised. Chitosan is studied because of its appealing properties and diverse applications. The purpose of this work is to synthesize chitosan nanoparticles (CSNPs) by ionic gelation method at various pH levels and test them against Mucor and other filamentous fungus. Field Emission Scanning Electron Microscope, Zeta sizer, Zeta potential, and Fourier Transformed Infrared Spectroscopy were used to characterize CSNPs. Hydrodynamic size increased considerably with increasing pH. Our CSNPs were tested against fungal isolates of *Aspergillus Flavus RCMB 02783, Aspergillus Fumigatus RCMB 02564*, and *Aspergillus Niger RCMB 02588, Penicillium Notatum (NCPF 2881)* and   (*M. circinelloides* CNRMA 03.894) *causing mucromycosis*. Antifungal activity was investigated using Minimum inhibitory concentration (MIC), Minimum Fungicidal concentration (MFC), Disc diffusion assay, and Antifungal inhibitory percentages methods. The best antifungal efficacy results were obtained through CSNPs prepared at pH = 4.4 at very low concentration for MIC (1.03 or 2.75 μg/mL) with 100% *M. circinelloides* inhibition followed by pH = 4.6 with MIC (73 or 208 μg/mL) and 93%  *M. cirecinelloides* inhibition %. Future usage of these materials in masks or wound dressing to avoid fungal infections, including mucormycosis following COVID-19, penicillium, and aspergillosis toxicity and infections.

**Graphical Abstract:**

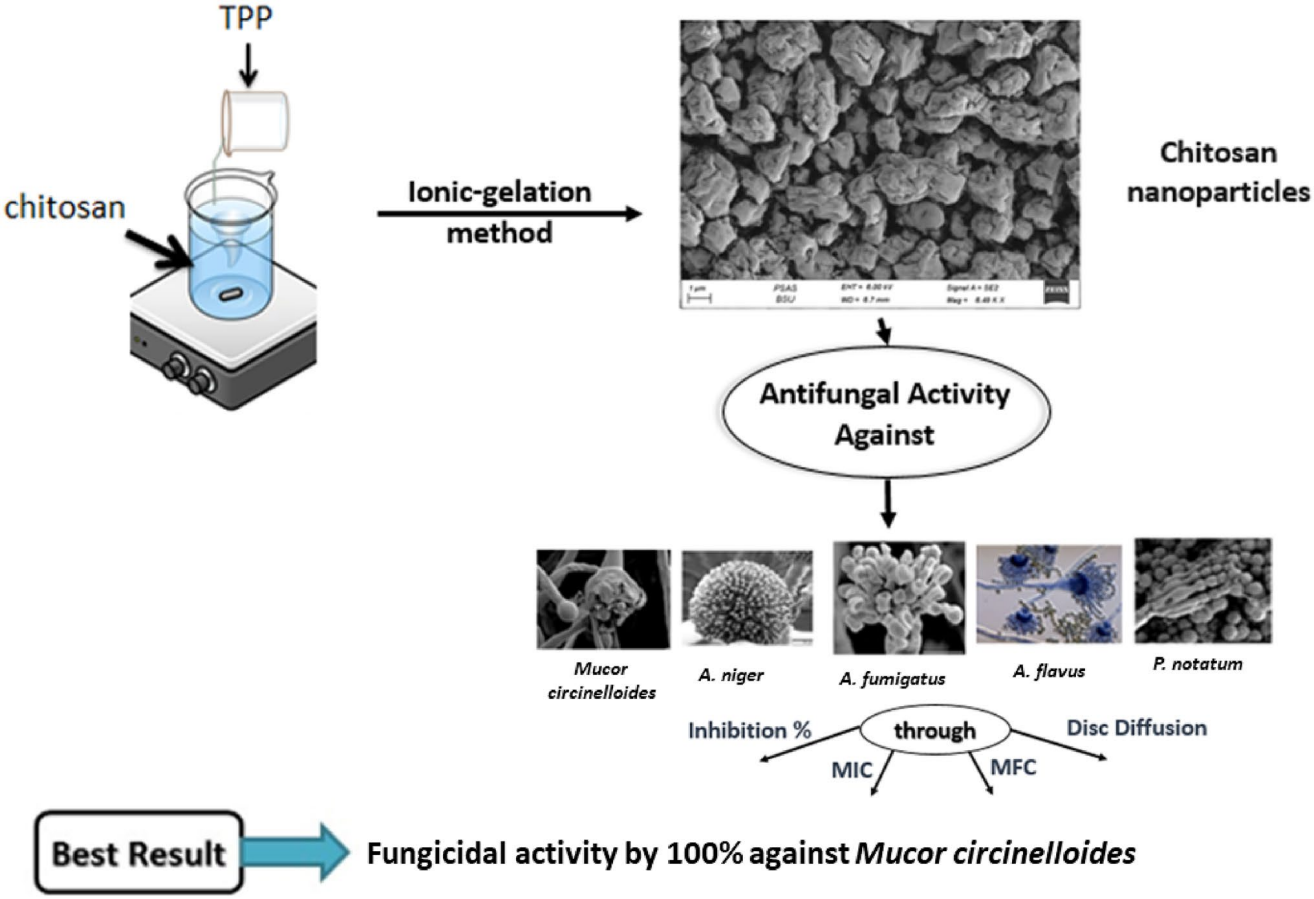

**Supplementary Information:**

The online version contains supplementary material available at 10.1007/s00284-023-03555-y.

## Introduction

Despite the limited availability of antifungal specialists, the fundamental cause of the usually increased toxicity linked to antifungal treatment is the close contact of fungus and mammalian cells [1]. Despite the revelation of wasted particles and the present availability of knowledge to minimize toxicity and boost bioavailability, the quest for underutilized antifungal specialists and the characterization of fresh targets continues [2]. A broad-spectrum and few adverse effects are necessary in a perfect antifungal agent [3]. Plants, animals, and terrestrial or marine creatures are abundant sources of potential antifungals due to their enormous metabolic capability [4] Hence, they are a rich source of beneficial chemicals [5]. Engineered fungicides are applied to crops to prevent comparable parasite infections that could affect human and animal health by contaminating soil and water [6]. Due to fungi's resistance to various commercial fungicides, there have been considerable financial losses [7]. This issue has prompted the search for untapped innovations and antimicrobial agents, including nanomaterials, to combat parasitic diseases in crops.

Chitosan is a polysaccharide derived from chitin, a biopolymer extracted from various organisms, most notably marine crustaceans [8]. Chitosan is a copolymer of two monomers, *N*-acetyl-d-glucose and d-glucose amine [9]. Due to its abundance, biodegradability, biocompatibility, non-toxicity, metal complexation, surface modification potential, and antimicrobial properties, it has garnered considerable interest. Chitosan has numerous applications in numerous fields, including cosmetics, agriculture, water treatment, environmental remediation, and medicine [10].

Although some filamentous fungi, particularly those belonging to the genera *Aspergillus* and *Penicillium,* such as *Aspergillus niger* and *Penicillium citrinum*, owing to the metabolites they produce, they are used in the food and pharmaceutical industries [11], Moreover, these fungi have been connected to diseases and infections [12]. It has been reported that some filamentous fungus can induce both invasive and superficial diseases, particularly in immunocompromised individuals with skin and nail infections [13]. Mucormycosis is a collection of fungal and contagious diseases and infections that can affect the skin and the gastrointestinal tract. Mucormycosis occurs when the organism comes into contact with an injured or compromised organism and rapidly multiplies.

On the other hand, mucormycosis is a very rare illness. It is caused by the fungus Mucor, which is often found in manure, decaying fruits and vegetables, soil, and plants. After COVID-19 infection, it may be deadly for diabetics or those with severely compromised immune systems, such as cancer patients or persons with HIV/AIDS, and it affects the sinuses, brain, and lungs. The use of corticosteroids, which is a life-saving treatment for COVID-19 patients who are very unwell, may produce mucormycosis, which has an overall mortality rate of 50%. Steroids reduce lung inflammation and raise blood sugar levels in both diabetic and nondiabetic COVID-19 patients, even though they appear to prevent some of the damage that the body's immunological response can cause. The outbreaks of mucormycosis are thought to be caused by this loss of immunity. Also, both *Penicillium* and *Aspergillus* species are filamentous fungi that cause fatal diseases.

We sought to shed light on the straightforward preparation of CSNPs at various pH values. In addition, the effect of particle size and preparation conditions on the antifungal activity against certain filamentous fungi was investigated.

## Materials and Methods

### Materials

Chitosan with a very low molecular weight (molecular weight > 10,000), derived from shrimp shells 75% (deacetylated), was obtained from LOBA CHEMIE PVT.LTD. Sodium tripolyphosphate (TPP) (anhydrous extra pure) was obtained from Oxford Lab Chem. LLP, along with glacial acetic acid, sodium hydroxide (NaOH), and bidistilled water. Hemocytometer chamber, Malt Extract broth, Oxoid Sabouraud’s dextrose agar (SDA), Sabouraud’s dextrose broth (SDB), cyclohexamide, and 0.8 M sorbitol.

### Preparation of Chitosan Nanoparticles

Chitosan nanoparticles were synthesized using the ionic gelation mechanism of TPP. Briefly, 1% (v/v) acetic acid was used to dissolve chitosan at room temperature with magnetic stirring until the solution became transparent. Next, TPP (1 mg/mL) was dissolved in distilled water during magnetic stirring, 100 mL of TPP was slowly added to an equal volume of chitosan solution, and the mixture was stirred gently for 3 h. A 0.1 M NaOH solution was prepared and added to the mixture drop by drop, and the pH was adjusted to 4.2 using a pH meter. The other samples were prepared using the same method but with pH values of 4.4, 4.6, and 4.8. The solutions were left to dry at 40 °C in a desiccator.

### Characterization of Chitosan Nanoparticles

The hydrodynamic size was determined by means of the dynamic light scattering method Zeta sizer (Malvern Nano-Zs90). In order to conduct the measurements, nano-chitosan was disseminated in 1% (w/v) acetic acid. The solutions were treated for 15 min in an ultrasonic bath. Using the same zeta sizer, the zeta potential of chitosan nanoparticles has subsequently been measured. Different magnification levels of field emission scanning electron microscopy (FESEM, Zeiss Sigma 500 VP Analytical FESEM, Carl Zeiss, Germany) were used to determine the morphology of the nanomaterials. The spectral region 4000–400 cm^−1^ was scanned with a VERTEX 70 (FT-IR) spectrometer coupled to a RAM || FT-Raman module in order to characterize the chemical bonds.

### Isolation and Identification of Strains

Fungal isolation; From fungal infected shrimp and clinically infected mastitis; three distinct fungal species (three *Aspergillus* isolates, *P. notatum* and *M. circinelloides*) were positioned in pre-enrichment broth (Malt Extract broth, Oxoid®) and incubated at 25 °C for 5 days, then refined on SDA (Oxoid®) and incubated for 3 days. These species were chosen due to their important significance clinical emergency effect in human and animal's life. The recovered fungi were identified morphologically in accordance with Rippon (1988). All the tested isolates were kindly provided by Al-Azhar University's Regional Institute for Mycology and Its Applications called Regional Center for mycology and biotechnology (RCMB) performed both morphological and molecular fungal identification using standard isolates for *M. circinelloides, P. notatum*, and three types of Aspergillus isolates*.* They also determined the mycelial organisms based on mycelial morphology, turn-around color, and lactophenol cotton blue recolored smear examination. Further biochemical research or laboratory testing, such as sugar fermentation and assimilation tests (glucose, maltose, galactose, and lactose), urease, and germ tube development, validated the results [14] at that mycology center. Regional Centre for Mycology and Biotechnology (RCMB) isolates for Aspergillosis included *Aspergillus Flavus* RCMB 02783, *Aspergillus Fumigatus RCMB 02564*, and *Aspergillus Niger RCMB 02588*, *Penicillium Notatum (NCPF 2881)* and *(M. circinelloides* (*CNRMA 03.894*) causing mucromycosis. The ITS region of CNRMA 04.805 (GenBank accession number DQ118990) of *M. circinelloides* was sequenced, and a BLAST search revealed complete identity (100%) in the ITS-1 and ITS-2 regions with the corresponding sequences available in the data bank from two reference strains.

### Fungal Isolates and Inoculum Preparations

Fresh cultures of *Aspergillus flavus* (*A. flavus*), *Aspergillus fumigatus* (*A. fumigatus*), *A. niger*, and *P. notatum* were utilized to make suspensions, which were plated on SDA and incubated for 72 h at 25 degrees Celsius. Following incubation, samples were transferred to test tubes containing 5 mL of 0.9% saline solution using a bent needle. A hemocytometer or automated cell counter was used to standardize the final inoculum's turbidity in order to count the number of spores. Liquid Yeast Peptone Glucose medium is frequently used for suspension cultures. The final concentration of freshly collected *M. circinelloides* spores was obtained at approximately 1.5 × 10^7^ colony-forming units per milliliter (CFU/mL) [15].

### Minimum Inhibitory Concentration (MIC) and Minimum Fungicidal Concentration (MFC) Determination

#### Minimum Inhibitory Concentration (MIC)

To estimate the minimal inhibitory concentration (MIC) of the investigated products, the broth microdilution method was used against *M*. *circinelloides*, *A*. *flavus*, *A*. *fumigatus*, *A*. *niger, and P*. *notatum* isolates used in the biological assays [15]. One hundred microliters (100 μL) of SDB A solution were dispensed into each well of a microdilution plate with 96 "U"-shaped wells. Next, 100 μL of the nanomaterials emulsion was put to the first horizontal row of wells on the plate. In double serial dilutions, a 100 μL aliquot was taken from the well with the highest concentration and transferred to the next well, resulting in concentrations ranging from 1000 to 1.9 μg/mL. In the end, 10 μL of an inoculum solution comprising various strains examined was put to each well of the plate, where each column represented a fungal strain. In the presence of the standard antifungal cyclohexamide with 500 mg/L media for prevention of rapid growing molds, a positive control (media with the materials but without fungal strains) and negative control (media without the materials and fungal strains) were grown (media with the fungi but without nanomaterials).

All CSNPs prepared at various pH levels were dissolved in acetic acid (1%). Consequently, 100 µL of SDB containing varied concentrations of tested chitosan nanoparticles in two fold serial dilutions was added to each well (1000, 500, 250, 125, 62.5, 31.25, 15.62, 7.81, 3.90, and 1.95 µg/mL) After diluting the NPs, 10 µL suspensions containing 1.5 × 10^87^ fungal strains/mL were inoculated; acetic acid served as the negative control.

At 25 °C, the plates were incubated for 72 h. The presence (or absence) of growth was visually observed after the proper incubation period. The MIC was established as the lowest concentration that visibly inhibited fungal growth while considering the formation of cell clusters or "buttons" in the plate wells. The studied nanoparticles' antibacterial activity was explained (considered active or not) based on the criteria outlined by Morales et al. [16]: strong/good activity (MIC: lower than 1000 μg/mL).

#### Minimum Fungicidal Concentration Assay

On SDA-coated Petri dishes, 1 μL aliquots of MIC, MIC × 2, and MIC × 4 of the tested nanomaterials, Cyclohexamide (500 mg/L), and the negative control for fungal growth were subcultured to determine the MFC. After 24–48 h of incubation at 35 °C, the MFC was evaluated based on its growth relative to the controls. The minimal fungicidal concentration (MFC) was then established as the lowest product concentration that inhibited the development of various fungal species and produced either 50% or 99.9% fungicidal activity [17]. Biological activity assays were performed in triplicate, and the results were expressed as the arithmetic means of the MIC and MFC concentrations. Subsequently, it was possible to determine whether the substance was active using both dilution methods. The MFC test was carried out since it was unclear if it would actually kill the fungus or just inhibit its growing. Depending on the tested fungus species, small aliquots of each broth dilution test were sub cultured on a rich solid medium and incubated for a predefined duration of time and temperature. According to Chemical and Laboratory Standard Institute-standardized publications. The MFC is recognized as the lowest concentration of the chemical at which observable subculture growth is not seen. Moreover, MFC might offer information on fungicide or fungistatic action. If MFC and MIC are same, the material is a compound fungicide; if MFC is greater than MIC, the substance is fungistatic [18].

### Sorbitol Assay-Effect CSNPs Prepared at Different pHs on the Cell Wall of Different Tested Fungal Strains

The test was done using sorbitol-containing and sorbitol-free (control) medium to examine putative antifungal processes involved in the impact of nanomaterials on the cell walls of several fungi. The culture media (peptone water medium) was supplemented with sorbitol at a concentration of (0.8 M sorbitol 5 g/L added on peptone water media 15 g/L). In "U"-shaped 96-well plates, the assay was conducted using the microdilution technique. On the fifth day of incubation, after the plates had been aseptically sealed and incubated at 35 °C, readings were obtained. Based on sorbitol's potential to operate as an osmotic protective agent for the fungal cell wall, the cell wall is one of the likely cell targets for the investigated nanomaterials, as shown by the elevated MIC values in the medium containing sorbitol compared to the normal medium. Cyclohexamide served as the standard antifungal medication. The experiment was conducted in triplicate, and the findings were reported as the arithmetic mean [19].

### Agar Diffusion Method

The agar diffusion method is a semi-quantitative experiment involving the addition of a sample with a known concentration (chitosan NPs produced at various pHs) to an agar surface that has previously been infected with a standard number of fungus cells. Disk diffusion, in which sterile filter paper discs (6 mm) are infected with the sample and then put to the agar surface, is one method for applying the sample. The samples diffused into the agar medium after inoculation, generating a circular concentration gradient. If the sample exhibited antifungal action, a zone of growth inhibition formed around the disc as the fungus proliferated. Some writers divide this zone of inhibition into three categories: total inhibition, moderate inhibition, and no inhibition [20].

### Agar Dilution Assay

The Agar Dilution Technique for Determining Chitosan Nanomaterials' Antifungal Efficacy. According to Jeff–Agboola et al. (2012), the antifungal activity of nanomaterials was tested against randomly selected fungal strains [21]. Following 72 h of development on SDA at 25 degrees Celsius, the examined fungi were adjusted to 1.5 × 10^8^ CFU in physiological saline (0.9% NaCl). After preparing and autoclaving SDA at 121 °C for 15 min and storing it at 55 °C, the tested nanomaterials were manufactured and mixed with SDA in accordance with the concentration test. The evaluated chitosan nanoparticles were synthesized at 1%, 2%, and 3% concentrations. The chitosan-agar medium (20 mL) was then put onto sterilized Petri plates and allowed to solidify. On the agar plates, equal volumes of the fungal suspensions were inoculated and speared. The plates were then incubated at 25 degrees Celsius for 72 h prior to evaluation on the fifth day of incubation.

### Sequence Analysis

The nucleotide sequences of the ITS region of different isolates are available from GenBank under accession numbers OQ438646:OQ438650. Sequences were assigned names Egypt/BSU-9 Egypt/BSU-13.

### Statistical Analysis

The data were reported as the standard deviation of the mean from the mean (S.E.M.). A one-way analysis of variance was employed to evaluate statistical significance (ANOVA). Using SPSS (version 20.0), Tukey's post-hoc test for multiple comparisons was then conducted (IBM SPSS Statistic 20.0, Armonk, NY, USA). P values less than 0.05 were statistically significant.

## Results

### Colonial Appearance and Microscopical Examination

*Aspergillus niger* exhibited the formation of fully developed colonies during a period of 2 to 6 days. The growth originally manifested as a yellow colony, which afterwards exhibited a black, punctuated surface due to the production of conidia. As the colony matured, it exhibited a distinct transformation, becoming a jet black and powdery appearance, while the opposite side retained its original buff or cream coloration. Under microscopic examination, *A. niger* displayed septated hyphae, elongated conidiophores that provided support for spherical vesicles, which in turn generated huge metulae and smaller phialides. These phialides were responsible for generating lengthy chains of brown rough-walled conidia. The process of sporulation included the whole surface of the vesicle.

The *A. flavus* isolates exhibited a somewhat quick growth rate, with colonies displaying a yellow–green coloration during a span of 1 to 5 days. The opposite exhibits a lack of color, ranging from a pale pinkish hue to a dull or deeper shade. Under microscopic examination, the vesicles had a spherical shape, while the phialides originated either directly from the surface of the vesicles or from a basic row of branches known as metulae, which were arranged in a biserate manner. The phialides produced clusters of yellow−orange elliptical or spherical conidia, which exhibited surface roughening as they matured.

*  A. fumigatus* exhibited a fast growth pattern, with a colony appearing over a span of 2 to 6 days. The colony had a fluffy to granular morphology, ranging in color from white to blue–green. The predominant visual characteristic seen in mature colonies undergoing sporulation was the presence of a powdered blue–green look. A distinguishing feature of *A. fumigatus* is the presence of conidiophores that are either short or elongated at a microscopic level, accompanied by a unique cell located at their base. Additionally, septate hyphae may be seen as another hallmark of this species. A large, spherical vesicle with phialides that are shaped like bottles and cover about half to two-thirds of its top surface was seen to emerge from the tip of the conidiophore. A columnar mass consisting of elongated chains of tiny (2 to 3 µm in diameter), spherical, rough-walled, green conidia was seen on the vesicle. Aspergillus fumigatus had a quick growth rate, with a colony appearing over a span of 2 to 6 days. The colony displayed a fluffy to granular texture and ranged in color from white to blue-green. The predominant visual characteristic seen in fully developed colonies undergoing sporulation was the presence of a powdered blue-green hue. A distinguishing feature of *A. fumigatus* was the presence of conidiophores that were either short or elongated, accompanied by a distinct cell at their base, as well as the presence of septate hyphae when seen under a microscope. A large, spherical vesicle with phialides shaped like bottles, which cover about half or two-thirds of its top surface, was seen to emerge from the tip of the conidiophore. A columnar mass was seen on the vesicle, consisting of elongated chains of tiny spherical conidia. These conidia had a diameter ranging from 2 to 3 µm and had a rough surface texture. Furthermore, the conidia seemed green in color. The serological studies were conducted at the Serology Unit of the Animal Health Research Institute, located in Dokki, Giza, Egypt. (Figure S1)**.**

### Molecular Characterization of Some Isolated Fungi

The polymerase chain reaction (PCR) technique was used to analyze a total of 12 fungal isolates, consisting of 3 isolates each from *Aspergillus niger, Aspergillus flavus, and Aspergillus fumigatus*. This analysis included the use of fungus-specific universal primer pairs, namely ITS1 and ITS4. All isolates that were chosen for analysis had positive results in the polymerase chain reaction (PCR) when subjected to ITS1 and ITS4 primers. The resulting amplified product had a size of 570 base pairs. (Figure S2).

### Sequence Analysis

The nucleotide sequences of the ITS region of different isolates are available from GenBank under accession numbers OQ438646:OQ438650. Sequences were assigned names Egypt/BSU-9 Egypt/BSU-13 (Table 1). Sequence analysis revealed that the PCR amplified region contains 18S rRNA gene partial sequence; ITS1, ITS2 complete sequences; and 28S rRNA gene partial sequence. BLAST search of the assembled sequences showed identity of isolates.

**Table 1:** The assigned sequence accession numbers

Spp. name	Sample name in gene bank	Accession numbers
*A. niger*	Egypt/BSU-11	(OQ438648)
*A. flavus*	Egypt/BSU-12	(OQ438649)
*A. fumigatus*	Egypt/BSU-13	(OQ438650)

## Characterization of Chitosan Nanoparticles

### Size Distribution and Zeta Potential

Hydrodynamic size is an essential defining characteristic of Nano suspensions. Table 2 illustrates chitosan nanoparticles size distribution prepared at various pH levels. It was discovered that the size distribution was extremely narrow, indicating the excellent dispersion of the synthesized nanoparticles and the solvent, as well as the homogeneity of the obtained size. Consequently, we believe that this preparation method is effective and easily reproducible under the aforementioned conditions. It was discovered that the hydrodynamic size increases with pH, from 58 nm at pH = 4.2 to 128.4 nm at pH = 4.8 (Table 2). Furthermore, we observed a large variation in the hydrodynamic parameter of the obtained nanoparticles, although the pH value changed only from 4.2 to 4.8. This result confirms that the synthesis condition, specifically the value, is crucial in determining the hydrodynamic size.

**Table 2:** The variation in hydrodynamic size and mean zeta potential of nanochitosan prepared at different pH values

pH	4.2	4.4	4.6	4.8
Hydrodynamic size (nm)	58.8	114.9	125.3	128.4
Mean zeta potential (+ve) (mV)	45	30	100	75

By measuring the surface charge of nanoparticles, Zeta potential is an essential technique for examining their stability as seen in table 2. . It was discovered that zeta potential increased from + 45 mV at pH = 4.2 to + 75 mV at pH = 4.8. The highest zeta potential value was found in the sample prepared at pH = 4.6. The high zeta potential values for the four samples indicate the stability of the NPs.

**Fig. 1:**
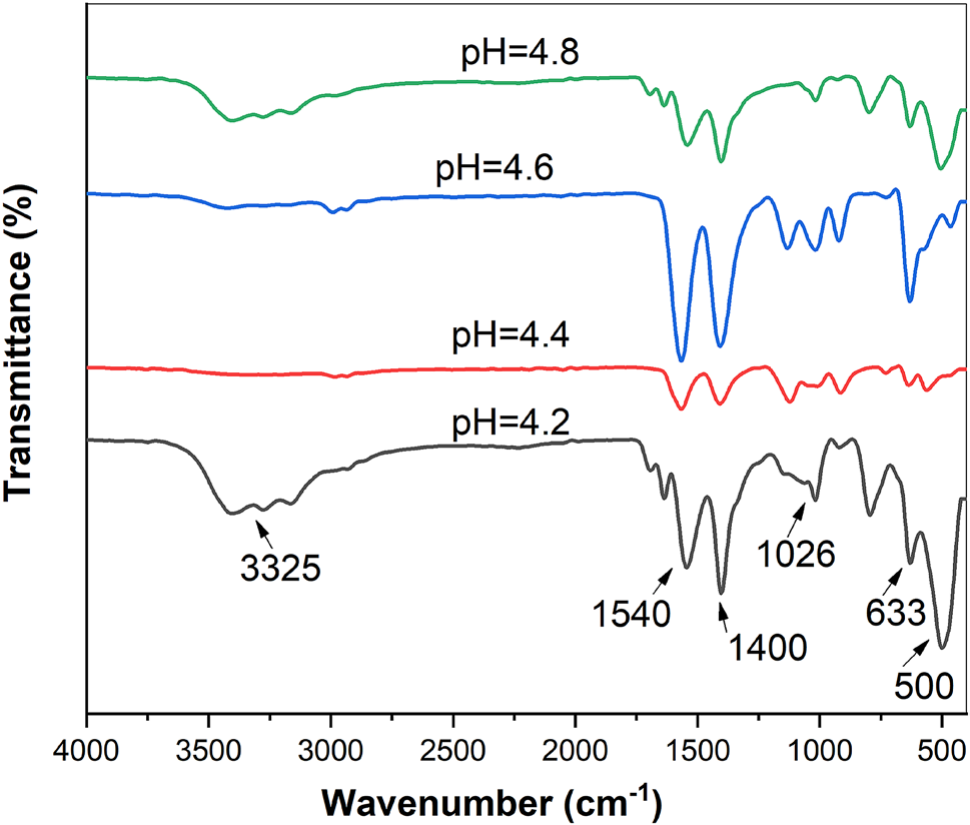
FT-IR spectra of nano chitosan prepared at **a** pH = 4.2, **b**  = 4.4, **c** pH = 4.6, **d** pH = 4.8

### Fourier Transformed Infrared Spectroscopy (FTIR)

The FTIR technique was used to investigate the functional groups present in prepared nanochitosan. Figure 1 displayed FTIR spectra that revealed a broad and intense peak at 3325 cm^−1^ due to the vibration of the –OH group. An absorption band at 1540 cm^−1^ was attributed to the N–H bend. At 1400and 1026 cm^−1^, two significant bands were found, and they were due to the vibration of C=O. The results were comparable to those of previous studies [22, 23]. As reported by Marei et al., normal chitosan has two absorption bands at approximately 1663^−1^ and 1618 cm^−1^ as doublet overlapped bands due to C=O stretching, whereas, in nanoscale, it is significantly altered [24].

### Field Emission Scanning Electron Microscopy (FESEM)

**Fig. 2:**
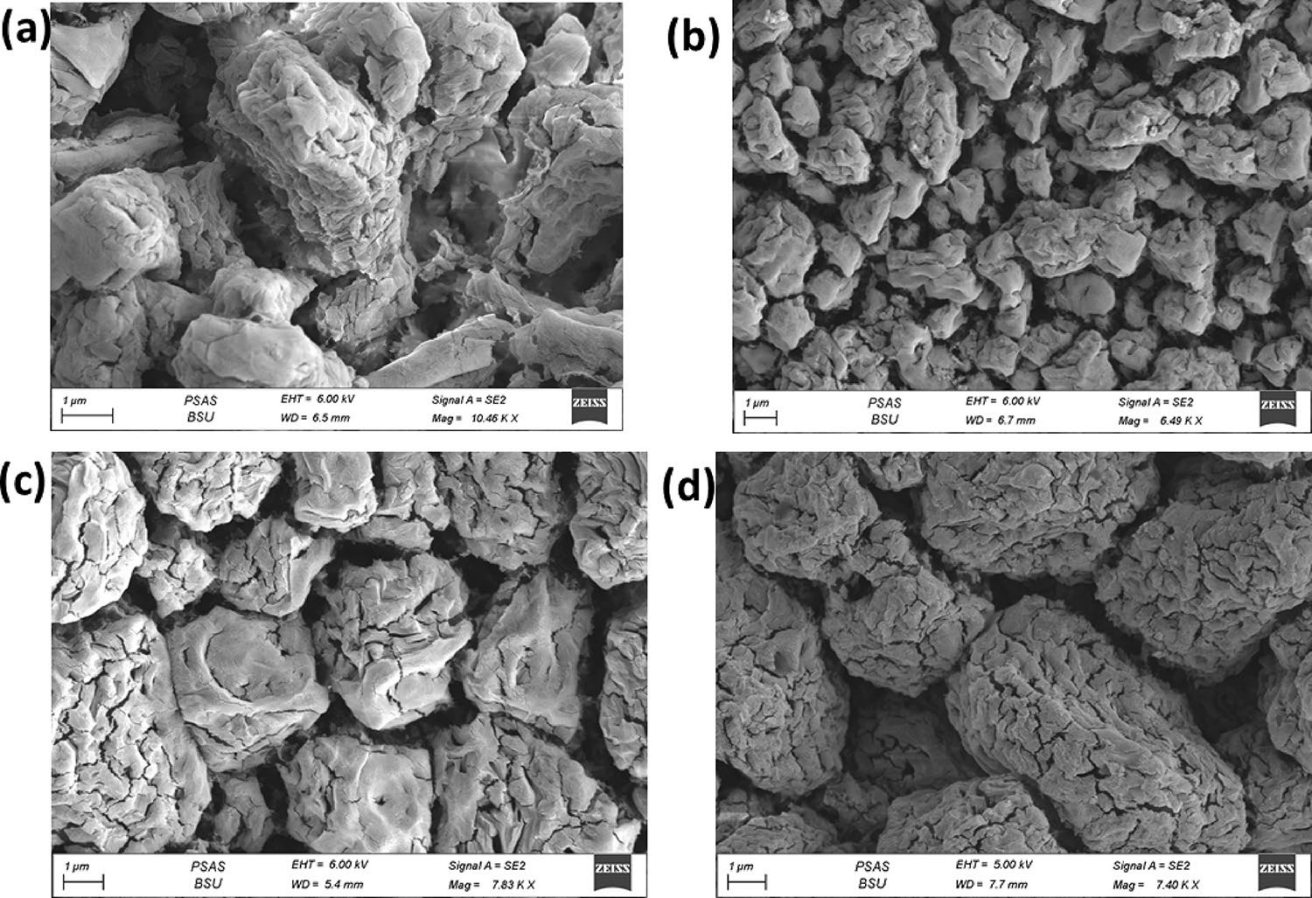
SEM images of nano chitosan prepared at **a** pH = 4.2, **b** pH = 4.4, **c** pH = 4.6, **d** pH = 4.8. which showed rock-like aggregates and or spherically shaped particulates

FESEM can reveal additional information regarding the morphology and structure of nanoparticles. Figure 2a demonstrates that chitosan nanoparticles show rock-like aggregates at pH = 4.2 and low magnification. Clusters of nano spherically shaped particulates are arranged in sheets that are ordered and stacked when viewed at a higher magnification. Mostly porous nano sheets were observed. The nanoparticles resemble sponge coral, which we can easily identify. By increasing the pH to 4.4, the rock morphology is kept unchanged and more as cracks form along the surface. As depicted in Fig. 2b, the sheets of nanospheres become more stacked, very close to one another, and of higher order when viewed from above under high magnification. The sample prepared at pH = 4.6 behaves similarly to that at pH = 4.4, but fractures occur in multiple directions. The nanospheres are arranged in wavy sheets in perfect order and homogeneous distribution, with neglected layer spacing. It is evident from Fig. 2c. At pH = 4.8, the aggregates have a longer length and a high volume of micro cracks. Importantly, further magnification revealed that the nanospheres are aligned in sharp-edged sheets of decreasing thickness. Figure 2d.

Due to their homogeneous morphology and large porosity, we could have recommended these nanoparticles for various applications, including water treatment and energy storage, based on FESEM studies.

### Broth Micro Dilution Method in Two Different Media

Numerous studies have also investigated how NPs interact with bacteria [25], but fewer have investigated their effects on fungi [26]. This may be as a result of the comparative simplicity of bacterial and fungal systems. One of the fungi most affected by chitosan nanoparticles, particularly those prepared at pH 4.4 and 4.6 at *p* value < 0.001 significantly than other testes pH values.

In all cases, the MICs were identical to the MFCs, indicating that the NPs possessed fungicidal properties. The MICs of chitosan nanomaterials prepared at various pH levels against different fungal strains were calculated. The pH 4.2 nanoparticles were more effective against all fungal species, especially *M. circinelloides* and *P. notatum* (MICs of 88 and 20 µg/mL, respectively; *p* < 0.001 in comparison to other testes pH). Moreover, in higher concentrations, or MIC (416 µg/mL), they demonstrated significant antifungal activity (*p* < 0.01) against additional species. The sample prepared at pH 4.4 was the most effective against *M. circinelloides* and *P. notatum* at (2.75 µg/mL, in addition to possessing excellent antifungal activity against *A. flavus* (31.6 µg/mL) at *P* < 0.001 when compared to other tested fungi or even in other different pH. *A. fumigatus, P. notatum*, and *M. circinelloides* were inhibited by chitosan nanoparticles at concentrations of 20, 104, and 208 µg/mL, respectively, *at P* < 0.01. A pH of 4.8 was effective against the *Penicillium* strain but at a higher concentration for the other fungal strains (*A. flavus, A. fumigatus*, *Mucor*, *and A. niger*) (500 µg/mL) *P* < 0.05. As shown in Table 3.

**Table 3:** MIC and MIC with Sorbitol (µg/mL) with Density of 1 × 10^7^ against *M. Circinelloides*, *P. Notatum, A. Flavus, A. Fumigatus* and *A. Niger* after 72 h incubation at 25 °C

Nano chitosan	MIC (µg/mL)					MIC/Sorbitol (µg/mL)				
pH	*M. Circinelloides*	*A.Flavus*	*A. Niger*	*A. Fumigatus*	*P. Notatum*	*M. Circinelloides*	*A* *Flavus*	*A* *Niger*	*A. Fumigatus*	*P* *Notatum*
(4.2)	88.33 ± 6.00	416.6 ± 14.3	416 ± 14	416 ± 14	20.5 ± 9.5^*^	36.36 ± 2.4^*^	166.66 ± 7.2	333.33 ± 14	333.33 ± 14	11.166 ± 6.6^**^
(4.4)	2.75 ± 1.29^**^	31.16 ± 0.2	62 ± 0.0	62 ± 0.00	2.75 ± 1.29^**^	1.033 ± 0.3^**^	15.33 ± 0.2	41.66 ± 1.7	41.66 ± 1.7	1.25 ± 0.00^**^
(4.6)	208.33 ± 0.7	500 ± 0.00	500 ± .00	20.5 ± 9.5^**^	104.16 ± 6.3	73 ± 4.7^*^	208.33 ± 7.2	333.3 ± 1.4	17.58 ± 1.2^**^	62.5 ± 0.00^*^
(4.8)	500 ± 0.00	500 ± 0.00	500 ± .00	500 ± 0.00	104.16 ± 3.6^*^	416.66 ± 1.4	333.33 ± 1.4	333.3 ± 1.4	333.33 ± 1.4	62.5 ± 0.00^**^
Reference (Cyclohexamide)	20.5 ± 9.5^**^	125 ± 0.9	125 ± 0.9	125 ± 0.9	35 ± 1.4^**^	10 ± 1.6^**^	66 ± 0.9	66 ± 0.9	66 ± 0.9	16.6 ± 3.9^**^

Mean ± SD

^*^Means *p* < 0.01

^**^Highly significance at *p* < 0.001 when compared to other fungal isolates in the same row for each tested material at specified *pH*

The experiment was performed, repeated, and measured using sorbitol to assess the antifungal activity of the nanomaterials tested. The MIC results were repeated in different sorbitol media to clarify the exact mechanism of action of the tested substances against various fungal strains, as a lower effective MIC indicates an effect on the fungal cell wall. After testing the MIC in different media with more added sorbitol, the results indicated that chitosan nanoparticles prepared at pH 4.2 demonstrated greater activity against *P. notatum, M. circinelloides*, *and A. flavus* at 11, 36, and 166 µg/mL, respectively, *at P* < 0.001, as opposed to the previously mentioned higher concentrations in normal MIC. The activity of the nanoparticles prepared at pH 4.4 was enhanced against *M. circinelloides, P. notatum*, and *A. flavus* strains (1, 1.25, and 15 µg/mL, respectively, *P* < 0.01). Against *A. fumigatus*, *P. notatum*, and *M. circinelloides*, the sample prepared at pH 4.6 exhibited greater activity with MIC values of approximately 17, 62, and 78 µg/mL (Table 3), *P* < 0.05. As an osmoprotectant, the sample synthesized at pH 4.8 exhibited superior activity against Penicillium compared to other fungal species at *P* < 0.001.

### Minimum Fungicidal Concentrations (MFC)

The identical MFC and MIC values for chitosan NPs synthesized at pH 4.2 indicate the fungicidal activity of the synthesized chitosan. The pH 4.2 sample exhibited good fungicidal activity against *P. notatum* and *M. circinelloides* (respectively, 26 and 93 µg/mL); *P* < 0.001. As shown in Table 4, the best fungicidal activity against *M. circinelloides* was obtained by the sample synthesized at pH 4.4 at (2.75 µg/mL), *P* < 0.001. *A. fumigatus* was the most affected strain at pH 4.6, followed by *P. notatum* and *M. circinelloides*, *P* < 0.01. At pH 4.8, the Penicillium strain was the most affected, *P* < 0.001 in comparison to other testes fungal isolates (Table 4).

**Table 4:** MFC and MFC with sorbitol (µg/mL) with density of 1 × 10^7^ against *M. Circinelloides*, *P. Notatum, A. Flavus, A. Fumigatus* and *A. Niger* after 72 h incubation at 25 °C

Chitosan	MFC (µg/mL)					MFC/Sorbitol (µg/mL)				
pH	*M. Circinelloides*	*A.Flavus*	*A. Niger*	*A. Fumigatus*	*P. Notatum*	*M. Circinelloides*	*A* *Flavus*	*A* *Niger*	*A* *Fumigatus*	*P* *Notatum*
(4.2)	93.33 ± 5.4	500 ± 0.00	500 ± 0.00	500 ± 0.00	26 ± 9.5^*^	41 ± 1.7^*^	166.66 ± 7.2	416 ± 14	416 ± 14	15 ± 0.00^**^
(4.4)	2.75 ± 1.29^**^	31.16 ± 0.2	62 ± 0.0	62 ± 0.00	2.75 ± 1.29^**^	1.033 ± 0.3^**^	15.33 ± 0.2^*^	41.66 ± 1.7	41.66 ± 1.7	1.25 ± 0.00^**^
(4.6)	208.3333 ± 5.7	833.3 ± 2.75	500 ± 0.00	31 ± 9.5^**^	25 ± 0.00	104 ± 3.6^*^	250 ± 0.00**	333.33 ± 1.4	23.08 ± 4.6^**^	104.16 ± 3.6^*^
(4.8)	500 ± 0.00	500 ± 0.00	500 ± 0.00	500 ± 0.00	104.16 ± 3.6^*^	416.66 ± 1.4	500 ± 0.00	416.66 ± 1.4	416.66 ± 1.4	104.16 ± 3.6^**^
Reference (Cyclohexamide)	20.5 ± 9.5^**^	125 ± 0.9	125 ± 0.9	125 ± 0.9	35 ± 1.4^**^	20 ± 3.4^**^	66 ± 0.9	66 ± 0.9	66 ± 0.9	32 ± 2.6^**^

Mean ± SD

^**^Highly significance at *p* < *0.001* when compared to other fungal isolates in the same row for each tested material at specified pH

^*^Means *p* < *0.01*

In addition to lower effective fungicidal concentrations against *M. circinelloides* and *P. notatum*, the MFC test results with different media containing added sorbitol concentrations revealed lower fungicidal concentrations*.* The sample was produced at a pH of 4.2. For Penicillium and Mucor, the MFC concentration decreased from (26 and 93 µg/mL), *P* < 0.01. Mucor and Penicillium*,* with the best fungicidal concentrations at *(*1 and 1.75 µg/mL), *P* < 0.001, were effective against *P. notatum* infections when the sample was prepared at pH 4.4, *P* < 0.01. The sample prepared at pH 4.6 had the highest fungicidal concentrations against the *A. fumigatus* strain, *P* < 0.001, whereas the sample prepared at pH 4.8 had the highest concentrations against the Penicillium strain, *P* < 0.001 when compared to others.

### Disc Diffusion Method

**Fig. 3:**
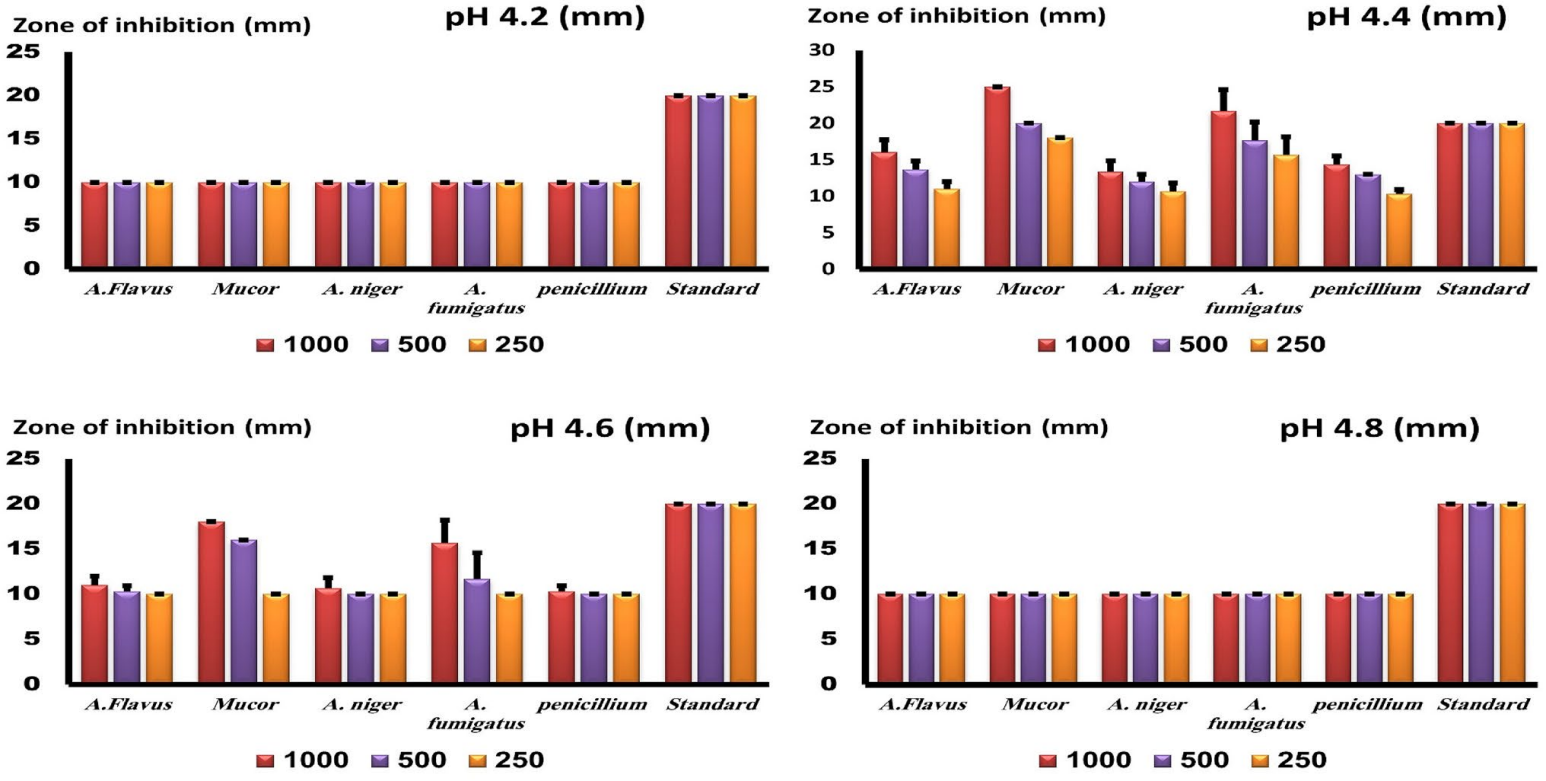
Zone of inhibition of nano chitosan synthesized at different pH in SDA media against density of 1 × 10.^7^ against *M. circinelloides*, *p. notatum, A. Flavus, A. Fumigatus* and *A. Niger* after 72 h incubation at 25 °C., measured as mean ± SD. Y axis represent the measured zone of inhibition in mm. The best zone of inhibition was obtained at pH 4.4 with significance *level p* < 0.001

The disk diffusion method is regarded as one of the most precise techniques for measuring antifungal or antibacterial activity. On SDA plates, zones of inhibition were measured on the SDA plates against different fungal strains, and multiple concentrations are depicted in Fig. 3. They were tested at concentrations (1000, 500, and 250 µg/mL); the sample was prepared at pH 4.2 chitosan showed a slight zone of inhibition against *M. circinelloides, P. notatum*, *and A. fumigatus* in comparison to the standard antifungal for rapidly growing fungi (chlorohexidine) at *P* < 0.01. Compared to the standard drug, nano-chitosan prepared at pH 4.4 produced the largest antifungal zone against the Mucor strain, *P* < 0.001. It also exhibited good antifungal activity against the other fungal strains. For samples prepared at pH 4.6 and 4.8, the optimal and largest zone against *M. circinelloides* and *A. fumigatus* strains were obtained, *P* < 0.001.

### Agar Dilution Assay (Percent of Inhibition %)

**Fig. 4:**
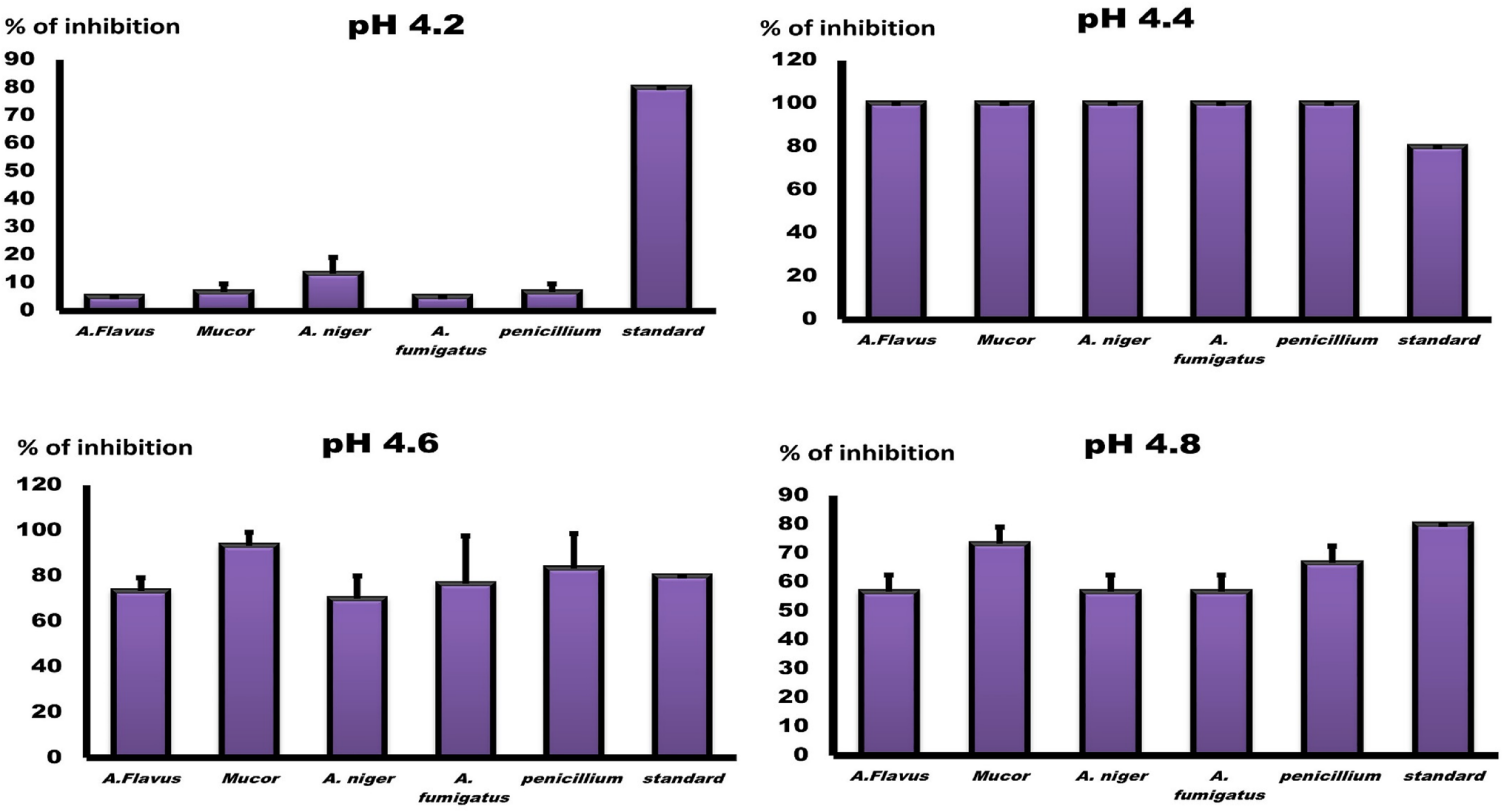
Percent of inhibition (%) of nano chitosan prepared at different pH in SDA media against density of 1 × 10^7^ against *M. circinelloides*, *p. notatum, A. Flavus, A. Fumigatus* and *A. Niger* after 72 h incubation at 25 °C, measured as mean ± SD. Y axis represent the percentage of antifungal inhibition (%). The best percentages of inhibition was obtained at *pH* 4.4 with significance *level p* < 0.001

**Fig. 5:**
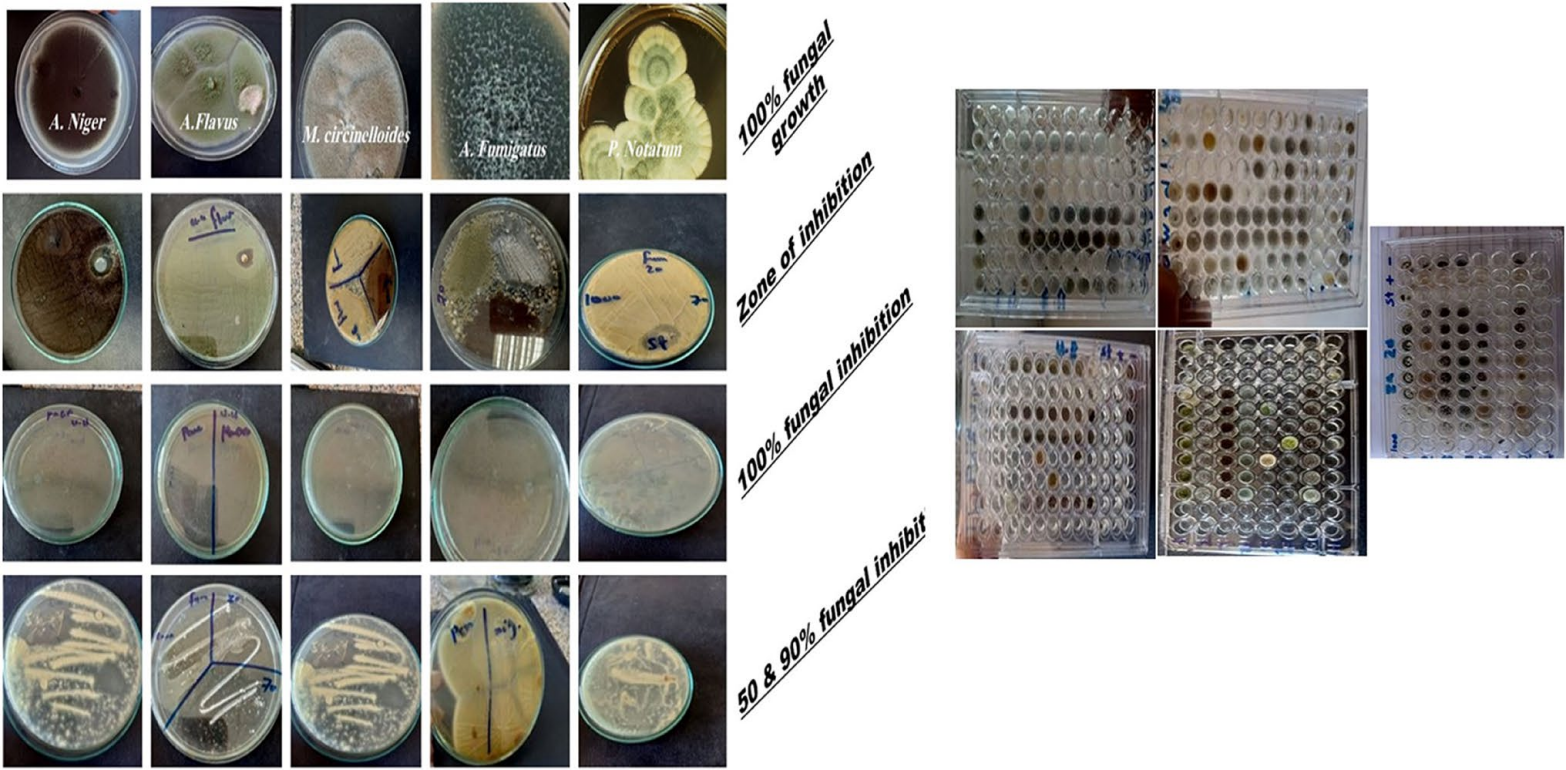
Shape of the different tested fungal isolates with 100% and zero growth besides zone of inhibition and inhibition percentage for nano chitosan against *M. circinelloides*, *p. notatum, A. Flavus, A. Fumigatus* and *A. Niger* filamentous fungi. While the U-shape plates are the MIC results (broth dilution test)

Based on the estimation of antifungal activity after the addition of materials to the media, the percentage of fungal inhibition was calculated and displayed in Fig. 4. At a pH of 4.2, chitosan nanoparticles exhibited moderate antifungal activity against *A. niger*, with 13% inhibition. Notably, those prepared at pH 4.4 demonstrated the greatest antifungal inhibition against all tested fungal strains (100%), more than the standard drug itself (80%) at *P* < 0.001. At pH 4.6, the best results were obtained against *M. circinelloides and P. notatum* (93 and 83%), *P* < 0.001 in comparison to other fungal isolates*,* whereas at pH 4.8, the results were 74 and 66%, *P* < 0.01. The antifungal activity of the samples results from their unique characteristics, such as small size, large surface area, and uniform distribution. The shape of the plate's zone of inhibition and MIC  U-shape plates were added as shown in Fig. 4 & 5.

## Discussion

*M. circinelloides* poses a significant public health risk. In immunocompromised people and animals, *M. circinelloides* is a common agent that causes Mucormycosis, a potentially fatal infection [27]. Depending on the location of the infection, *Mucormycosis*-related mortality rates can reach 96% [28]. It can contaminate and spoil food, resulting in foodborne illnesses such as food poisoning [29].

The size of nanoparticles is affected by several factors. This includes chitosan concentration and molecular weight [30], in this study, we demonstrate chitosan activity against the life-threatening Mucormycosis that accompanied COVID-19 pandemic infections or against additional fungal species, using different preparation methods and *pH*s.

Previous research has demonstrated that the effectiveness of chitosan depends on the type of fungus as well as the formulation of chitosan. Furthermore, three different fungal species were used to examine the effect of particle size or zeta potential on antifungal activity. The fungus *C. albicans* infects the skin and mucous membranes of humans. It may enter the bloodstream and disperse throughout the body [31]. In contrast, *Fusarium* species are commonly recognized as the causative agent in human opportunistic infections [32]. The most frequent agent responsible for food contamination is *A. niger.* Moreover, although uncommon in humans, high concentrations of *A. niger* can cause aspergillosis[33]. This study demonstrated that *A. niger* is extremely resistant to chitosan. In previous studies, only chitosan solution and nanoparticles made from high-concentration (high molecular weight) HMW chitosan was effective in inhibiting the growth of this fungus. This finding corresponds with research showing that just chitosan with a high molecular weight was more effective at inhibiting *A. niger* [34]. According to Allan and Hadwiger, fungi with chitosan as a part of their cell walls are more resistant to chitosan that has undergone external modification [35]. Both the presence of chitosan and 10% chitin in the cell wall of *A. niger* may explain its tenacity [36].

In conclusion, nanoparticle antifungal activity is influenced by particle size, distribution, and zeta potential. Different nanoparticles with different particle sizes or zeta potentials may inhibit fungi through distinct mechanisms. Chitosan's antifungal activity can be influenced by variables such molecular weight, polymerization degree, pH, and temperature. In our study, the effects of pH value during chitosan preparation affected both hydrodynamic size and antifungal activity. Understanding the primary mechanism of chitosan as an antifungal substance depends on the fact that chitosan is poly-cationic in nature; therefore, the inhibitory effect is due to chitosan adhering to negatively charged cell membrane components. (i.e., proteins, phospholipids). This may help to explain why other researchers' findings indicate that chitosan has an impact on the contagious layer and causes the release of a cellular substance [37]. Once followed, it maintains a distance from the diverse structures of the organism that proceed to form, causing more or less restraint based on the species of the life form as they fragment the transport of solutes into the cell [38].

In addition, Antifungal action may be attributable to the presence of phenolic chemicals that become active or increase when chitosan is added [31] who demonstrated an increase in peroxidase (POD) in the presence of chitosan, fungal strain growth is inhibited [39], In chitosan treatments, the levels of PPO (Polyphenol oxidases), POD, and phenolic compounds increased. Liu et al. observed that chitosan treatments raised the levels of polyphenol oxidases (PPO), polyphenol oxidase dehydrogenase (POD), and phenolic compounds. Another significant aspect of chitosan’s effect is that it is pH-dependent. As chitosan was diluted in acetic acid, its concentration decreased. It caused a massive denature of proteins. The outcomes of these studies indicate that the solvent used to dilute chitosan affects its antifungal activity [40].

Moreover, the negatively or adversely charged plasma film is, arguably, the most likely target location for poly-cationic molecules [41]. Hence, poly-cationic chitosan with a high surface charge will interact with the fungus more effectively. Furthermore, chitosan has a higher affinity for binding to fungal cells. Nano-sized chitosan contributes to a larger surface area; it enables nanoparticles to adhere more tightly to the surface of fungal cells, thereby compromising membrane integrity [42]. According to a study conducted by Ma and Lim, the cellular uptake of chitosan is high [43]. This indicated that chitosan may be able to diffuse into fungal cells and thereby inhibit both DNA and RNA synthesis. This could explain why chitosan nanomaterials have enhanced antifungal properties. Increasingly more has been discovered about the mechanism of action of chitosan; it precipitates and stacks on the surface of microbial cells, whose physiological pH is close to neutral. The formation of an impermeable layer will block the cell surface channels and prevent the passage of crucial nutrients needed for microorganism survival. Therefore, through the diffusion of chitosan nanoparticles into fungal cells, DNA or RNA synthesis is inhibited, resulting in the direct cell death of fungal cells.

Sorbitol, an osmotic protector, is utilized to maintain the stability of protoplasts in fungus. Some fungal cell wall inhibitors are distinguished by their ability to reverse antifungal activity in sorbitol-containing medium [44]. In the presence of inhibitors of the fungal cell wall, cells protected by sorbitol may proliferate, but growth would be impeded in the absence of sorbitol. The drop in MIC value obtained in media containing sorbitol as compared to media without sorbitol indicates this impact (standard medium) [44]. Osmotic destabilizing agents and rupture of the cell wall result in the rearrangement of the cell wall, which allows fungal cells to survive [45]. The examined chemicals seemed to have an effect on the cell wall, altering its composition, blocking its formation, and resulting in cell death, as well as reducing spore germination, proliferation, and respiration.

Similar to other ubiquitous fungal infections, infectious Mucor species induces immunosuppression that predisposes to Mucormycosis (particularly delayed and excessive neutropenia, a serious haematological condition with or without stem cell transplantation, and the delayed use of corticosteroids) [28]. Further risk factors include iron overload, therapy with the iron chelator deferoxamine, and poorly controlled diabetes mellitus with or without diabetic ketoacidosis [46]. The foremost common course of the disease is the inward breath of fungal spores, coming about in aspiratory or rhino-orbital-cerebral shapes of Mucormycosis. Diseases caused by the direct inoculation of Mucorales spp. After a major injury, such as burn wounds when the tissue has been injured directly, spores have been observed to spread into the damaged tissue [47], In these instances, infections can occur without other risk factors. In rare instances, spores entered the body through minor injuries, such as insect bites or animal scratches [48]. There have also been cases of gastrointestinal Mucormycosis, especially from spore ingestion [49]. A significant characteristic that all types of Mucormycosis have in common is the invasion of blood vessels, which leads to thrombosis and eventual tissue necrosis. The frequently observed spread of infection in Mucormycosis is also explained by angio-invasion. Mucormycosis is distinguished from other form diseases, such as obtrusive aspergillosis [50]. by its unique histopathological changes, its rapidly dynamic nature, and the widespread tissue rot that frequently accompanies it. Moreover, diabetes mellitus, press over-burden, and deferoxamine treatment are unique risk factors for mucormycosis. Due to the danger posed by this strain of fungus, it is essential to search for novel antifungal substances that can treat or prevent this type of severe fungal infection.

Based on our findings, *A. niger* was the most resistant strain of fungi. *A. niger*, in contrast to other fungal strains, was found to be extremely resistant to chitosan. In this work; the tested MIC; in media with more added sorbitol, the results showed that chitosan nanoparticles prepared at pH 4.2 showed more activity against *M. circinelloides, P. notatum*, *and A. flavus* with 11, 36, and 166 µg/mL, respectively, instead of the previously mentioned higher concentrations in normal MIC and in previous literature as higher concentrations were required to demonstrate good antifungal activity as; the MIC of chitosan in previous studies were at *C. albicans* (*LMW* 0.25–0.86 mg/mL and at *HMW* = 0.6–1.0 mg/mL) and *F. solani* (*LMW* = 0.86–1.2 mg/mL and *HMW* = 0.5–1.2 mg/mL). These findings indicated the efficacy of the prepared formulas for chitosan as antifungal activity at very low concentrations in micrograms and against life-threatening fungal strains when compared to previous studies at higher concentrations in milligrams. The activity of the nanoparticles prepared at pH 4.4 was enhanced against *M. circinelloides, P. notatum*, *and A. flavus* strains (1, 1.25, and 15 µg/mL), respectively). Moreover, the sample prepared at pH 4.6 displayed greater activity with lower MIC values of approximately 17, 62, and 78 µg/mL, respectively, against *A*. *fumigatus*, *Penicillium*, and *Mucor.*

## Conclusion

In the present work, CSNPs were successfully synthesized and characterized. According to our results, the pH of chitosan nanoparticles greatly affected their shape, particle size, zeta potential, and antifungal activity. Positive surface charge of the generated CSNPs enhanced their antifungal activity by interacting with negatively charged biological membranes. This research found that CSNPs had potent antifungal action against a range of fungal strains and that the amounts of nanomaterials that inhibit growth are identical to those that cause death. The best obtained antifungal efficacy results was obtained through chitosan prepared at pH = 4.4 at very low concentration for MIC (1.03 or 2.75 µg/mL) with 100% inhibition for *M. circinelloides* and also for all other tested fungal isolates followed by pH = 4.6 with MIC (73 or 208 µg/mL) and 93% of *M. circinelloides* inhibition and from 70–75% inhibition for the other fungal isolates. In comparison to the standard control positive drugs which was showed 80% of inhibition against all fungal isolates including the *M. circinelloides*. At MFC; the best results for Nano chitosan prepared at pH 4.6 was obtained against *A. Fumigatus* followed by *M. circinelloides*. While sample prepared at pH 4.4.the best MFC was against *M. circinelloides*. Based on our results, we suggest use of these materials especially, nano-chitosan prepared at pH = 4.4 in masks or wound dressing to prevent fungal infections, such as Mucormycosis after COVID-19, penicillium, and aspergillosis toxicity and infections. Future respective are required for detailed mechanism of action with more advanced investigations about the antifungal activity are important.

### Supplementary Information

Below is the link to the electronic supplementary material.

Supplementary file1 (DOCX 1119 kb)

## Data Availability

Being available after acceptance and publishing in this journal but not in open access form.
