# Supplementary material for: Antifungal Activity of Chitosan Polymeric Nanoparticles and Correlation with Their pH Against Mucor circinelloides Causing Mucormycosis, Along with Penicillium notatum and Aspergillus Species
Source: Curr Microbiol. 2023 Dec 22;81(1):47. doi: 10.1007/s00284-023-03555-y (PMC10746780; doi:10.1007/s00284-023-03555-y)
Supplement: Supplementary file 1 — Supplementary file1 (DOCX 1119 kb) [file 284_2023_3555_MOESM1_ESM.docx]

**Journal of Current Microbiology**

**Antifungal Activity of Chitosan Polymeric Nanoparticles and Correlation with their pH against *Mucor circinelloides* causing Mucormycosis, along with *Penicillium notatum* and Aspergillus species**

**Fatma Ibrahim Abo El-Ela^1^, Walid Hamdy Hassan^2^, Alaa M. Amer^3^, S. I. El-Dek^4^**

**Affiliations**

**Fatma Ibrahim Abo El-Ela^1^,** Assistant Professor of pharmacology, Department of Pharmacology, Faculty of Veterinary Medicine, Beni-Suef University, Beni-Suef, Egypt, 62511. [fatma.aboel3la@vet.bsu.edu.eg](mailto:fatma.aboel3la@vet.bsu.edu.eg) – [fa.pharma@yahoo.com](mailto:fa.pharma@yahoo.com)

**Walid Hamdy Hassan^2^,** Professor and Head of Bacteriology, Mycology and Immunology Department, Faculty of Veterinary Medicine, Beni-Suef University, Beni-Suef, Egypt, 62511. [walidhamdyhassan@yahoo.com](mailto:walidhamdyhassan@yahoo.com) - whh@bsu.edu.eg

**Alaa M. Amer^3^,** Material Science and Nanotechnology Department, Faculty of Postgraduate Studies for Advanced Science, Beni-Suef University, Beni-Suef, Egypt, 62511. [aalaamohsen6698@gmail.com](mailto:aalaamohsen6698@gmail.com) - aalaamohsen9001_sd@psas.bsu.edu.eg

**S. I. El-Dek^4^,** Material Science and Nanotechnology Department, Faculty of Postgraduate Studies for Advanced Science, Beni-Suef University, Beni-Suef, Egypt, 62511. [samaa@psas.bsu.edu.eg](mailto:samaa@psas.bsu.edu.eg)

**Corresponding author**

[**Fatma I. Abo El-Ela**](https://sciprofiles.com/profile/author/SFM1aU52NEhLUDJxVkpBaWloL3hSemtIWnRBYmxHNUNnNFYvUjlRdWVZTT0=): Assistant Professor of Pharmacology, Department of Pharmacology, Faculty of Veterinary Medicine, Beni-Suef University, 62511, Egypt. [Fatma.aboel3la@vet.bsu.edu.eg](mailto:Fatma.aboel3la@vet.bsu.edu.eg) [fa.pharma@yahoo.com](mailto:fa.pharma@yahoo.com) +2 01007248079

**
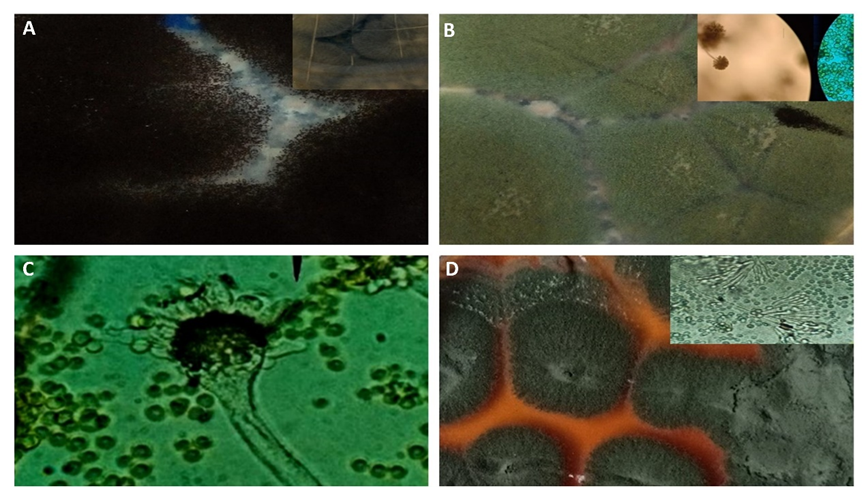
**

**Figure S1: Colonial appearance and Microscopical investigation of some tested isolates**

**
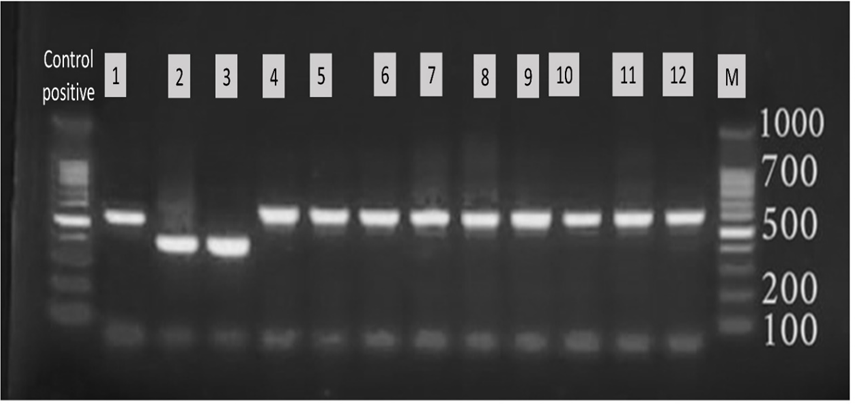
**

**Figure S2:** **Molecular characterization of some isolated fungi. PCR results for 12 fungal isolates. Amplification of 570bp fragment of ITS region from all tested isolates.**

**+Ve: Control positive (*A. niger*). M: 100bp DNA ladder.**

**1: *C. albicans* 7&9: *A. flavus***

**2&3: *C. neoformans* 8: *A. flavus***

**4: *A. niger* 10&12: *A. fumigatus***

**5&6: *A. niger* 11: *A. fumigatus***
